# Inhibition of trypsin expression in *Lutzomyia longipalpis *using RNAi enhances the survival of *Leishmania*

**DOI:** 10.1186/1756-3305-2-62

**Published:** 2009-12-09

**Authors:** Mauricio RV Sant'Anna, Hector Diaz-Albiter, Murad Mubaraki, Rod J Dillon, Paul A Bates

**Affiliations:** 1 Vector Group, Liverpool School of Tropical Medicine, Liverpool, UK; 2 Biomedical and Life Sciences, Lancaster University, Lancaster, UK

## Abstract

**Background:**

*Leishmania *parasites must overcome several barriers to achieve transmission by their sand fly vectors. One of the earliest threats is exposure to enzymes during blood meal digestion. Trypsin-like enzymes appear to be detrimental to parasite survival during the very early phase of development as amastigotes transform into promastigote stages. Here, we investigate whether parasites can affect trypsin secretion by the sand fly midgut epithelium and if inhibition of this process is of survival value to the parasites.

**Results:**

Infections of *Lutzomyia longipalpis *with *Leishmania mexicana *were studied and these showed that infected sand flies produced less trypsin-like enzyme activity during blood meal digestion when compared to uninfected controls. RNA interference was used to inhibit trypsin 1 gene expression by micro-injection into the thorax, as trypsin 1 is the major blood meal induced trypsin activity in the sand fly midgut. Injection of specific double stranded RNA reduced trypsin 1 expression as assessed by RT-PCR and enzyme assays, and also led to increased numbers of parasites in comparison with mock-injected controls. Injection by itself was observed to have an inhibitory effect on the level of infection, possibly through stimulation of a wound repair or immune response by the sand fly.

**Conclusion:**

*Leishmania mexicana *was shown to be able to modulate trypsin secretion by *Lutzomyia longipalpis *to its own advantage, and direct inhibition of trypsin gene expression led to increased parasite numbers in the midguts of infected flies. Successful application of RNA interference methodology to *Leishmania*-infected sand flies now opens up the use of this technique to study a wide range of sand fly genes and their role in the parasite-vector interaction.

## Background

*Lutzomyia longipalpis *sand flies are vectors of visceral leishmaniasis in South America [1]. Traditionally known as a disease of rural communities, visceral leishmaniasis has become increasingly widespread and urbanised, with approximately 74% of Brazilian states recording indigenous cases in recent years [2]. *Lu. longipalpis *is also an important and widely used permissive experimental host, capable of being infected with a wide range of *Leishmania *species under laboratory conditions [3]. Both sexes are plant feeders, but only adult female flies transmit disease, they ingest blood from mammals to obtain the protein necessary for egg production and maturation. During an infective blood meal, *Leishmania *amastigotes from an infected mammalian host are ingested with the blood and are exposed to digestive proteolytic enzymes, facing a complex and biochemically hostile environment inherent to blood digestion. The amastigotes transform into promastigote forms in the midgut of the female sand fly, subsequently undergoing a complex developmental cycle [4]. Serine proteases (including trypsin-like) are the most important digestive enzymes in the midgut of Dipteran blood-sucking insects [5]. The possibility that insect-derived digestive enzymes might play a role in influencing the development of medically important pathogens has been considered in other insect vectors. For example, in *Plasmodium gallinaceum*-infected *Aedes aegypti*, an *Aedes *trypsin was identified as the activating enzyme of a *Plasmodium *chitinase essential for ookinetes to escape the peritrophic matrix and establish the infection [6]. Similarly, the use of soybean trypsin inhibitor in dengue-2 virus-infected *Aedes aegypti *resulted in slower virus replication in the midgut, possibly due to inhibition of proteolytic processing of DENV-2 proteins [7]. In contrast, RNAi knockdown of 5G1, a serine protease associated with late phase digestion in *Aedes*, significantly increased midgut infection with dengue-2 virus [8].

In phlebotomine sand flies, previous work regarding the effect of proteolytic enzymes on *Leishmania *survival has been conducted using various parasite-vector combinations. The infection of *P. papatasi *with *Le. major *promastigotes was found to reduce midgut trypsin and chymotrypsin-like activity, suggesting a parasite modulation of those digestive enzymes [9]. Importantly, amastigote-initiated infections of *Le. major *i.e. using the correct life-cycle stage, also caused a significant suppression of alkaline protease, trypsin and aminopeptidase activity in the midgut of *P. papatasi *[10]. Evidence for the suppression of protease activity was also seen in *Le. major*-infected *P. langeroni*, a sympatric but unnatural vector [10]. Conversely, addition of soybean trypsin inhibitor to the blood meal enabled *Le. donovani *to develop in *Phlebotomus papatasi*, a vector usually refractory to this *Leishmania *species [9,11]. These reports suggested that a failure to reduce trypsin-like activity might be detrimental to parasite development in some way. This idea received further support in studies showing that parasites (*Le. major *in *P. papatasi *and *Le. mexicana *in *Lu. longipalpis*) exhibited a "window of vulnerability" to trypsin-like activity during amastigote to promastigote transformation [12,13]. Further, disruption of formation of the peritrophic matrix increased parasite mortality, whereas enhancement of this process protected the parasites [12,14], effects ascribed to increased or decreased exposure to trypsin-like activity secreted from the midgut epithelium, respectively. Although the biochemical basis of amastigote resistance to trypsin remains unknown, it is likely that expression of lipophosphoglycan by promastigotes protects these stages against such proteolytic attack [15].

Previous reports have identified 4 trypsin-like transcripts in *Lu. longipalpis *with high sequence similarity to *P. papatasi *midgut trypsins [16,17]. *Lu. longipalpis *trypsin 1 expression is blood meal-induced, peaking at ~12 hours post-blood meal, is the major trypsin-like activity and is absent in unfed flies, whereas trypsin 2 is constitutively expressed but at a low level [16]. In the current report, we test the hypothesis that *Leishmania *infections result in the modulation of trypsin-like activities in *Lu. longipalpis *to help the establishment of parasites. To this end we have used the RNAi technique employed for studies of gene function in various insect species and vector-parasite interactions [18,19], and recently developed by ourselves for use in phlebotomine sand flies [20]. This study is also the first to combine the RNAi technique with *Leishmania *infection in phlebotomine sand flies. We show that *Lu. longipalpis *trypsin 1 knockdown by RNAi has a positive effect on *Leishmania *survival in the sand fly midgut.

## Methods

### Insects

A laboratory colony of *Lu. longipalpis *established from sand flies caught in Jacobina (Bahia, Brazil) and kept at the Liverpool School of Tropical Medicine was used in all experiments and maintained using standard methods [21]. Insects were provided with 70% sucrose *ad libitum *and were reared under controlled conditions of temperature (27 ± 1°C) and humidity (80-95%). Adult females were fed on hamsters or rabbit blood twice a week. All procedures involving animals were performed in accordance with UK Government (Home Office) and EC regulations.

### Double stranded RNA synthesis

The trypsin 1 template was PCR amplified based on the *Lu. longipalpis *trypsin 1 gene sequence described before (GenBank accession number EF011106; ref 16) using a plasmid preparation obtained from a normalized *Lu. longipalpis *cDNA Library [22], and the ampicillin resistance gene (AMP; used as a control) was PCR amplified from the pBluescript SK plasmid (Stratagene). PCR was carried out using specific primers containing the T7 site at their 5 prime ends (Table 1). The PCR products of 551 bp for Tryp1 and 534 bp for AMP were used as templates for transcription reactions using the Megascript RNAi Kit (Ambion^®^) according to the manufacturer's instructions. After synthesis, column purification and elution with RNAse-free water at 65°C, dsRNA purity was determined by 1.5% agarose/ethidium bromide gel electrophoresis, and dsRNAs were quantified using a Nanodrop ND-1000 Spectrophotometer. Double stranded RNA samples were concentrated to 0.45, 2.5 and 4.5 μg/μL prior to use using a Christ^® ^RVC 2-25 rotational vacuum concentrator and stored at -80°C until needed.

**Table 1:** Primer sequences for dsRNA construction (DS-) and RT-PCR (RT-)

Primer	5'-3' sequence	Size (bp)
DS-Trypsin 1 F	TAATACGACTCACTATAGGGAGAGTCTTCACCGCTGCCCATTG	551
DS-Trypsin 1 R	TAATACGACTCACTATAGGGAGATCGCGAACGGAAGAAACACG	
		
DS-AMP F	TAATACGACTCACTATAGGGATGCCGGGAAGCTAGAGTAAGTA	534
DS-AMP R	TAATACGACTCACTATAGGGAAACGCTGGTGAAAGTAAAAGATG	
		
RT-Trypsin 1 F	GTCTTCACCGCTGCCCATTG	423
RT-Trypsin 1 R	GTCCTTGCCGCCCTCTTCA	
		
RT-Trypsin 2 F	GGAGGCAAGCCCGTGAGCATC	553
RT-Trypsin 2 R	CAACGACGGGACCGCCAGAAT	
		
RT-Lulong 60S L3 F	TCTCATCGGAAGTTTTCTGC	850
RT-Lulong 60S L3 R	GGCTTGTGACACCCTTGAAT	

### Double stranded RNA microinjections

Double-stranded RNAs were microinjected as described before [20]. Briefly, one day old, unfed *Lu. longipalpis *females were briefly chilled on ice, placed on an ice pack under a dissecting microscope and injected with 32 nanolitres of 0.45, 2.5 or 4.5 μg/μL stock dsRNA solution (14.4, 80 and 144 ng dsRNA per fly, respectively) for *Lu. longipalpis *trypsin 1 (knock down group) or dsAMP (4.5 μg/μL - mock-injected group) in the mid-lateral thorax, using a Drummond^® ^Nanoject II Auto-Nanolitre injector. After injections, flies were kept in a cage (>90% relative humidity) and supplied with 70% (w/v) sucrose solution *ad libitum*. Double stranded RNA-injected sand flies were fed with fresh rabbit blood heated to 35°C, through a chick skin feeder 72 h after injection and used for trypsin knockdown analysis and/or infected with *Le. mexicana*. Uninjected sand flies of the same age were used as a second control group.

### RNA extractions and RT-PCR to verify trypsin 1 knockdown by dsRNA injection

Total RNA was extracted from individual whole bodies of *Lu. longipalpis *using the RNaqueous 4-PCR Kit (Ambion^®^). Sequences of primers for RT-PCR are given in Table 1 and was performed using the AccessQuick RT-PCR System (Promega^®^) according to the manufacturer's instructions. RNA templates were normalized to 10 ng/μL and RT-PCR done using 24 cycles. RT-PCR was carried out using individual flies from each experimental group. Expression of the trypsin 1 gene in *Lu. longipalpis*, which were injected with trypsin 1 or AMP dsRNAs, were evaluated alongside expression of a housekeeping gene (AM088777, 60S ribosomal protein L3). RT-PCR products were analysed by 1.5% agarose/ethidium bromide gel electrophoresis and reduction in trypsin 1 gene expression was determined by densitometric measurement of bands using the software GeneSnap/GeneTools (Syngene, UK). Changes in gene expression in dsTryp1 and dsAMP-injected flies were determined by the ratio between band intensity of the trypsin 1 and corresponding 60S L3 products (loading controls). Analysis of expression was also done by RT-PCR for the trypsin 2 gene previously described for *Lu. longipalpis *(LlTryp2 - GenBank accession number EF011107; [16]).

### Trypsin enzyme assay

Trypsin assays were performed essentially as previously described [23]. Briefly, Benzoyl-arginine-*p*-nitroanilide (BApNA) was prepared as a 2 mM stock solution in 7% dimethylformamide (DMF) in distilled water. Midgut preparations (20 μL - supernatant from one gut homogenised in 100 μL 0.15 M NaCl) were mixed with 80 μL of 0.2 M Tris buffer (pH 8.5) and 80 μL 2 mM BApNA (final concentration 1 mM) in the well of a microtitre plate at room temperature (~22°C). The rate of reaction was measured automatically by linear regression using a microplate reader (VersaMax, Molecular Devices) at 405 nm for 5 minutes. One unit of specific trypsin activity was defined as the amount of enzyme which catalyses the formation of 1 μMol of product per minute per milligram of total protein by reference to a standard curve for *p*-nitroanilide.

### Protein assay

The total amount of protein in the whole body was quantified essentially as described [24]. Briefly, a 5 μL volume of *Lu. longipalpis *whole body homogenate in 0.15 M NaCl was mixed with 200 μL of the BIORAD^® ^Protein assay reagent (diluted to working concentration in distilled water) and endpoint absorbance was measured in a 96 well plate with a microplate reader (VersaMax Microplate Reader, Molecular Devices Inc.) at 595 nm. Bovine serum albumin was used as a standard.

### Parasite preparation and sand fly infection with Leishmania mexicana

Frozen aliquots of *Le. mexicana *amastigotes in 10% DMSO were rapidly thawed from liquid nitrogen and gently mixed with 10 mL Grace's insect medium (Sigma), supplemented with 25 μg/mL gentamycin sulphate and 20% heat inactivated FBS, pH adjusted to 5.5. The amastigotes were centrifuged at 1500 g for 10 min and washed twice with 10 mL of complete Grace's medium, before re-suspension in 10 mL of complete Grace's medium. Parasites were transferred into a culture flask at a final concentration of 2 × 10^5 ^parasites per mL, and cultured at 32°C as axenic amastigotes for one week. To infect sand flies, axenic amastigotes were resuspended in 1.5 mL of rabbit blood to a final concentration of 2 × 10^6^parasites per mL and were offered to female *Lu. longipalpis *through a chick-skin membrane. Flies were maintained at 27°C and 80-95% humidity with free access of the flies to 70% sucrose solution until required for experimental analysis The potentially infected midguts were removed by dissection and transferred to 1.5 ml microfuge tubes containing 50 μL of 0.15 M NaCl. Parasite counts were performed using a haemocytometer chamber.

### Statistical analysis

When data was normally distributed, ANOVA was used for multiple comparisons to test for significant differences between groups, and a pair-wise t-test was used to test for differences between means of two independent groups. When the data distribution was non-normal, the Kruskal-Wallis was used for multiple comparisons and the U Mann-Whitney test for differences between medians of two independent groups. All data was analysed using SPSS v 17.0.

## Results

### Infection of Lu. longipalpis with Le. mexicana reduces trypsin-like activity in the sand fly midgut

**Figure 1:**
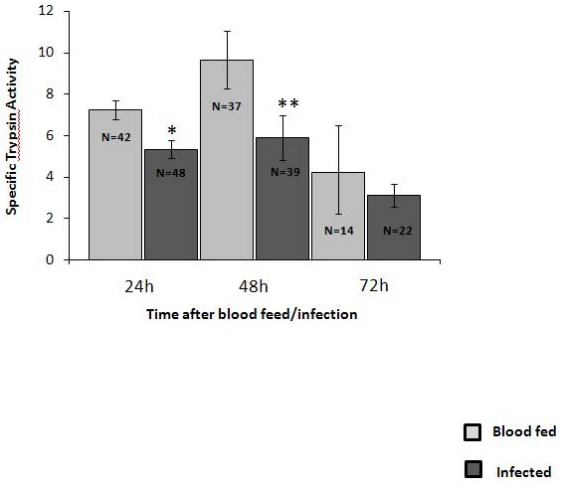
**Specific trypsin activity in the midguts of *Lu. longipalpis***. Sand flies were fed with rabbit blood alone or infected with *Le. mexicana *and trypsin activity (μM/min/mg protein) assayed at 24, 48 and 72 h after the blood feed/infection. Bars represent mean values and standard errors of combined samples derived from 2 independent experiments. Numbers of flies assayed are as indicated by N values. * represents statistical significance at *p *= 0.002 and ** represents statistical difference at *p *= 0.015 (*U *Mann-Whitney).

Female *Lu. longipalpis *were infected with *Le. mexicana *amastigotes and the trypsin activity in the midgut assayed at various times post-infection (Fig. 1). Compared to blood fed controls (sand flies fed with rabbit blood alone), infected flies showed a significantly reduced specific trypsin activity at both 24 and 48 hours after infection, and an overall reduction of ~30% in enzyme activity. These results show that feeding a *L. mexicana *amastigote infected bloodmeal results in a modulation of trypsin-like activity within the midgut of *Lu. longipalpis*.

### Injection of dsRNA for Lu. longipalpis trypsin 1 induces gene-specific knockdown

**Figure 2:**
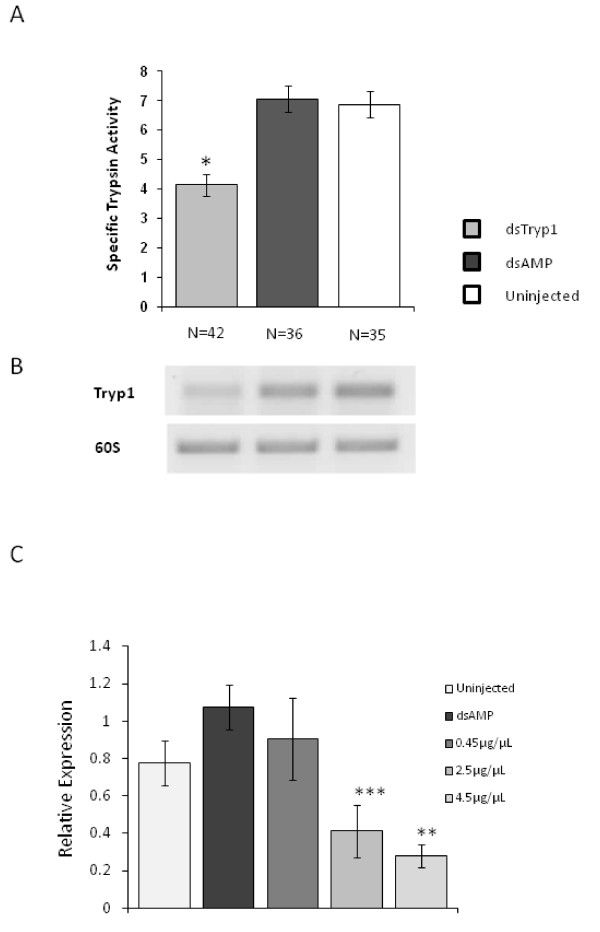
**Effect of dsRNA injections on trypsin 1 expression**. A. Reduction of specific trypsin-like activity in the midgut of dsRNA Trypsin 1-injected *Lu. Longipalpis *compared to dsAMP-injected and uninjected sand flies. Bar charts represent mean ± SEM of samples combined from 3 independent experiments.* indicates statistical significance at *p *< 0.05 (*U *Mann-Whitney). B. Representative RT-PCR showing individual sand fly profiles of the *Lu. longipalpis *Trypsin 1 gene for dsTryp1-injected, dsAMP-injected and uninjected sand flies. C. Relative expression of the *Lu. longipalpis *Trypsin 1 gene for uninjected sand flies, mock-injected flies, and dsTryp1-injected sand flies using stock concentrations of 0.45, 2.5 and 4.5 μg/μL (14.4, 80 and 144 ng of dsRNA per fly, respectively). Bar charts represent mean ± SEM of samples combined from 2 independent experiments. Kruskal-Wallis *p *= 0.013. ** indicates statistical significance at *p *= 0.016 between sand flies injected with 4.5 μg/μL dsRNA compared to uninjected, mock-injected and flies injected with 0.45 μg/μL. *** indicates statistical significance at *p *= 0.032 between flies injected with 2.5 μg/μL and mock-injected flies (*U *Mann-Whitney).

Double stranded RNAs encoding a 551 bp portion of the trypsin 1 gene of *Lu. longipalpis *(full length 768 bp) and ampicillin resistance gene (control dsRNA) were synthesized by PCR using suitable primers with the T7 site at their 5' ends (Table 1) and prepared to a final concentration of 4.5 μg/μL. Female sand flies were microinjected with dsRNA for the trypsin 1 gene and mock-injected with dsRNA for the ampicillin gene as previously described [20]. Following dsRNA injection, a 40% reduction in specific trypsin enzyme activity could be observed 24 hours after the blood meal in sand flies injected with dsTryp1 in comparison with mock-injected and uninjected *Lu. longipalpis *(Fig. 2A; *p *< 0.05). The effect of dsRNA injection on trypsin 1 mRNA expression was also investigated by RT-PCR. For example, Figure 2B shows trypsin 1 gene profiles for three individual sand flies used in the enzyme assays, confirming reduction in trypsin 1 gene expression upon microinjection.

To optimise the trypsin 1 knockdown 3 different stock concentrations of dsTryp1 were injected (Fig. 2C). Flies were microinjected with 32 nl of solutions containing 0.45, 2.5 or 4.5 μg/μL corresponding to 14.4, 80 or 144 ng of dsRNA per fly, respectively. An inverse correlation between dsRNA concentration and trypsin 1 mRNA relative expression was observed. Sand flies injected with 4.5 μg/μL dsRNA showed significantly reduced relative expression in comparison with groups of insects injected with 0.45 or 2.5 μg/μL, those mock-injected with dsAMP at 4.5 μg/μL and uninjected *Lu. longipalpis *(*p *= 0.013, Kruskal-Wallis;*p *= 0.016, U Mann-Whitney). Relative expression was also significantly reduced in flies injected at 2.5 μg/μL when compared to mock-injected sand flies (*p *= 0.032). The results showed that the knockdown effect was concentration dependent. The maximum knockdown was at the highest dsRNA concentration tested of 4.5 μg/μL, and this concentration was used in all subsequent experiments. Attempts to use higher concentrations were unsuccessful because of the high viscosity of the dsRNA solution.

### Trypsin 1 knockdown by RNAi increases parasite load in the midgut of Lu. longipalpis

**Figure 3:**
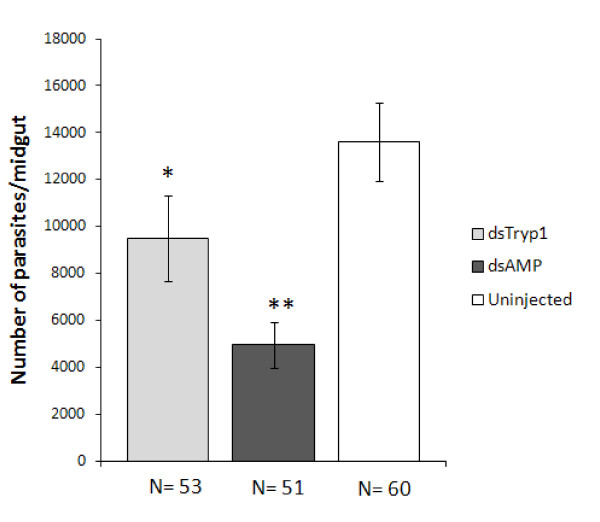
**Effect of trypsin 1 knockdown on *Le. mexicana *populations in the midgut of *Lu. longipalpis***. Bar charts represent mean parasite numbers ± SEM of data pooled from 5 independent experiments. Kruskal-Wallis *p *= 0.002. * represents statistical significance at *p *= 0.035 between dsTryp1 and dsAMP-injected sand flies and ** represents statistical significance at *p *= 0.001 between uninjected and dsAMP-injected sand flies (*U *Mann-Whitney).

Confirmation that dsRNA trypsin 1 injection reduced trypsin 1 mRNA expression and trypsin activity in the midgut of *Lu. longipalpis*, led to investigation of the impact of trypsin 1 knock down on parasite survival within the midguts of infected flies. Sand flies were microinjected with dsRNA for the trypsin 1 gene or mock-injected with dsRNA for the ampicillin resistance gene (control group) prior to infection with *Le. mexicana*. A further group of uninjected sand flies was also included in these infection experiments to determine if the injection process itself had any influence on the development of the infection. Double stranded RNA-injected *Lu. longipalpis *were kept in cages for 72 h after the injections with access to 70% sucrose solution via cotton wool. At day 3 post injection, 2 × 10^6 ^*Le. mexicana *amastigotes were mixed with rabbit blood and offered to dsRNA-injected sand flies, along with age-matched uninjected control flies. Only fully engorged blood-fed flies were used for subsequent analysis, these being kept for a further 72 h to allow infections to develop, prior to dissection of their midguts and estimation of the mean number of parasites in the gut (Fig. 3). Injection with dsRNA for trypsin 1 yielded infections with significantly higher numbers of parasites, approximately twice, compared to the mock-injected dsRNA AMP-injected sand flies (*p *= 0.035). Thus knockdown of trypsin 1 was of clear survival benefit to the parasite. The number of parasites in the uninjected group was higher than in both the injected groups. This difference was not statistically significant in those flies injected with dsRNA trypsin 1, but in the dsRNA AMP-injected sand flies the parasite numbers were approximately 2.7 fold lower than in the uninjected group (*p *= 0.001). This indicates that an anti-parasitic response of some kind had been triggered by injection, possibly by mechanical injury. However, apparently injection with dsRNA for trypsin 1 could rescue this anti-parasitic effect, as the number of parasites in sand flies injected with dsRNA for the trypsin 1 gene was not significantly different.

### Effect of trypsin 1 knockdown on trypsin 2 gene expression

**Figure 4:**
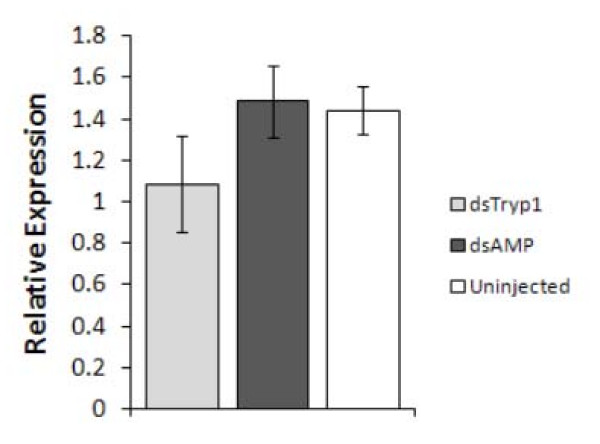
**Relative expression of the *Lu. longipalpis *early trypsin 2 gene in response to trypsin 1 knockdown**. Sand flies were uninjected, mock-injected or dsTryp1-injected with the concentration of 4.5 μg/μL (144 ng of dsRNA per fly). Bar charts represent mean ± SEM.

The four *Lu. longipalpis *trypsin genes described so far show high similarity between each other at the amino acid level [17] and a considerable similarity in their nucleotide sequence (Fig. S1 see Additional File 1). The most highly expressed transcript during blood feeding is trypsin 1, with trypsin 2 next (and trypsins 3 and 4 expressed at low levels). To investigate whether it might be affected by dsRNA injections for trypsin 1, the expression of trypsin 2 was determined by RT-PCR using primers specific for this gene (Fig. 4). The results show that there was no significant difference in trypsin 2 mRNA levels. Therefore, there is no evidence for the existence of a potential cross knockdown effect between these two genes, the biologically significant effects of trypsin 1 knockdown were confined to the homologous gene, this also being the major blood meal induced trypsin-like activity in the sand fly midgut.

## Discussion and Conclusion

*Leishmania *parasites are strictly confined to the midguts of their vectors during their development [25] unlike some other protozoan parasites transmitted by insect vectors such as *Plasmodium*. Therefore, survival of *Leishmania *parasites in phlebotomine sand flies is strongly associated with factors related to blood meal digestion: digestive enzymes released in the midgut after blood feeding that can modulate parasite growth [9,11,26], the peritrophic matrix that can act as a barrier to digestive enzyme dissemination and parasite migration to the anterior part of the midgut [12,14], and binding to the epithelial cells of the midgut to avoid being passed out with blood-derived excretions [27]. In this study we have shown that the reduction in trypsin activity in infected sand flies reported previously in *Phlebotomus *spp. [9-11], is also replicated in *Lu. longipalpis *upon infection with *Le. mexicana*, indicating that this is probably a general effect in *Leishmania*-infected sand flies. The main trypsin activity in recently blood fed flies is trypsin 1, being strongly upregulated in response to blood feeding and expressed at much higher levels than any of the other trypsin genes (trypsin 2, 3 or 4) [16,17]. Enzymatically it is not currently possible to distinguish between these enzymes (using specific substrates), but we can deduce that most, if not all of the reduction in trypsin activity caused by the parasite is due to down-regulation of trypsin 1 expression. Given that biochemical inhibition of trypsin-like enzymes with soybean trypsin inhibitor also aids survival of parasites [12,14], an ability to modulate midgut trypsin activity would be of clear advantage to the parasite. Either delaying [23] and/or reducing overall trypsin levels (without compromising host viability) until the parasites had fully transformed to protease-resistant procyclic promastigotes would be of selective advantage and a trait that would be strongly favoured by natural selection. The biological relevance of this effect is supported by earlier studies in which 50-80% of parasites were killed by trypsin during early bloodmeal development [12,13]. Under natural conditions where the numbers of ingested amastigotes will be much lower than in such experimental infections the advantage to the parasite is likely to be significant. However, it is interesting to note that a complete inhibition of fly trypsin activity would likely have longer term undesirable effects for the parasite, such as reducing available nutrients or compromising fly survival. Thus a balance probably exists where the level of inhibition induced maximises the overall transmission of the parasite, this likely to vary in different parasite-vector combinations. The mechanism behind this down-regulation effect is currently unknown, but others have also found evidence for the ability of the parasite to modulate sand fly gene expression during early bloodmeal digestion, including transcripts for trypsin genes [17]. Possible explanations include the release or secretion by amastigotes or transforming parasites of some "factor" that can influence the secretion of trypsin by the midgut epithelium. In the current study we have only examined trypsin activity, since this is the only sand fly enzyme currently known to exert a direct antiparasitic effect. However, previous work has shown that at least one other enzyme (aminopeptidase) is also down-regulated by infection [10], and it is possible that other proteases or different classes of enzyme could also be affected. Although these various data are persuasive, all of the evidence for *in vivo *antiparasitic effects of trypsins is indirect. Therefore, in the current study we sought to directly inhibit the expression of midgut trypsins using the RNAi approach.

Successful *Lu. longipalpis *trypsin 1 gene knockdown was achieved through dsRNA microinjections into the thorax of female sand flies as measured at both the mRNA and protein (enzyme activity) levels. Since the original publication of specific gene knockdown through dsRNA [28], gene knockdown by RNAi has become a powerful tool to study gene function in many different organisms [29]. Consequently this reverse genetic technique has also been applied to vector research and, for example, is currently being used to study many different aspects of vector-parasite interactions in mosquitoes [8,18,19,30-33]. A full genome sequence programme for *Lu. longipalpis *and *P. papatasi *is currently under way, various cDNA libraries have already been created [16,17,22] and RNAi-mediated gene knockdown has now been successfully achieved in *Lu. longipalpis *for two different genes [[20], current report]. Therefore, it is now possible to exploit this technique to study the functions of a wide range of genes in phlebotomine sand flies.

Knockdown of *Lu. longipalpis *trypsin 1 expression was shown to be of direct survival benefit to the parasite, the number of parasites in these flies being approximately double that in mock-injected controls. This result supports the hypothesis that secretion of trypsin into the midgut lumen has an adverse effect on the early phase of parasite development in the sand fly as previously reported [12,14] and that modulation of midgut trypsin helps establishment of the parasites in the gut. Both amastigotes and procyclic promastigotes are known to be relatively trypsin-resistant [12], but the reasons why transforming parasites should be relatively susceptible remain uncertain. However, it is known that the repertoire of surface proteins, glycoproteins and glycolipids is radically different between these two life cycle stages [34] and that they are adapted to survive at different temperatures [35], so there is a major turnover of *Leishmania *surface molecules during the transformation of amastigotes to promastigotes. Studies using mutants of *Le. major *and *Le. donovani *defective for surface lipophosphoglycan or phosphoglycans in general (LPG1 and LPG2 mutants, respectively) showed that parasite protection against midgut enzymes seemed to occur via phosphoglycan-containing molecules [15]. The ability of *Leishmania *to survive and develop during early stages of blood meal digestion can be considered the first major hurdle for successful establishment of the parasite population within the sand fly vector [27].

An interesting and unexpected finding in the current study was the effect of injection itself on the subsequent development of *Leishmania *infections; this caused a significant reduction in parasite numbers compared to uninjected controls. The observations that trypsin 1 knockdown was able to abrogate this effect and rescue the infection was impressive. However, it leads to the question of why injection of an unrelated control dsRNA should have mediated such an effect. The most likely explanation is that the mechanical injury caused by the injecting needle stimulated an immune/repair response in the flies, and that a byproduct of this was to reduce the number of parasites. The mechanism remains to be determined, but support for this interpretation comes from previous study with *Lu. longipalpis *[36] in which, alongside an increase in the humoral response associated with *E. coli *and *M. luteus *injections into the haemolymph, an increase in anti-bacterial activity was observed following control injections with culture medium alone when compared to uninjected controls. Further, it has been shown in *P. dubosqci *that injection with bacteria or infection with *Leishmania *parasites up-regulated expression of a sand fly defensin [37], and infection of *Lu. longipalpis *with *Le. mexicana *exacts a fitness cost on the sand flies [38]. Therefore, it is plausible that the act of pricking the cuticle of *Lu. longipalpis *during injections induced an anti-parasitic response in the sand fly, reflecting in reduction of parasite numbers in the midgut. The injection procedure is not sterile and, therefore, haemolymph contamination with bacteria cannot be ruled out. Alternatively, the injection of substantial amounts of dsRNA may have caused a physiological stress response that culminated in the induction of an immune response against the parasite. Whatever the precise mechanism, these observations support the existence of an interplay between the immune response of the sand fly and *Leishmania *infection that requires further investigation.

## Competing interests

The authors declare that they have no competing interests.

## Authors' contributions

MRVS, RJD and PAB designed the study. MRVS, HD, MM and RJD performed the experimental work. MRVS, HD, RJD and PAB analysed the data. MRVS, RJD and PAB prepared the manuscript, which was read and approved by all the authors.

## Supplementary Material

Additional file 1 Figure S1. Click here for file
