# Supplementary material for: Inhibition of trypsin expression in Lutzomyia longipalpis using RNAi enhances the survival of Leishmania
Source: Parasit Vectors. 2009 Dec 9;2:62. doi: 10.1186/1756-3305-2-62 (PMC2796656; doi:10.1186/1756-3305-2-62)
Supplement: Additional file 1 — Figure S1. [file 1756-3305-2-62-S1.DOC]

**A**

241 300

**Tryp2** ATCCTTAGTG AAAGGTTCAT CCTTACGGCA GCTCACTGTA TCATTGG-CG CAACTC--CT

**Tryp4** ATCATCAGTG ATAAATTCAT CTTGTCTGCA GCTCACTGTT ATATTGGTCG CACTGC--CT

**Tryp1** ATCGTTTCCC ACAACTTTGT CTTCACAGCT GCCCATTGCA CTGATGGCCA AGATGCTTCA

**Tryp3** CTCATAAGTG AATCATTCGT TCTCACAGCA GCTCATTGCA CGTTTGGTTC AAGTGCAAAT

301 360

**Tryp2** GATTTCACAG TCAGAACTGG ATCAAACTAC AGCTCAACAG ACGGTGAGGT CCATAAGGTG

**Tryp4** -TTTTCCAAG TAAGAGTTGG TTCGACAAAT AGCACTGCTG GTGGGACACT TTACACAGTT

**Tryp1** CACCTAAAGG TGCGAGTTGG ATCGAACGAA CACGGAGCTG GAGGAGATTT CTTCAAGGTT

**Tryp3** AGCTTCACGG TGCGTACACA GACGAGCTTC CACGGGAGAG GTGGTGTGGT GGTTGGTGTT

361 420

**Tryp2** CGACAGATCA TCAGCCATGA ACTCTACGAC GAGGAAACCA CGGATTACGA CTTCAGTATC

**Tryp4** AAAGATATCC ATGCCCATAC GTCCTACAAT GATACCACAT ATGACTACGA TTTTAGCATC

**Tryp1** AAGAAGGTTC ATCAGCATCC ATTATTCAAC TACCAAACTG TGGACTATGA CTTCTCCCTT

**Tryp3** AAGAGGATCA TTCAACATCC CAAATTCGAC TACTCAACCA TCGATTATGA TTTCTCAATT

421 480

**Tryp2** TTTGAACTGG AAGATCCCAT TAC---GTTC AGTGATACAA GGAGGGCAGT TCAACTTCCC

**Tryp4** TTTGAATTAA ATGATGCGAT TGCTCTGGAT AATGTTACTA GTCGTGTTGT GAGACTTTCT

**Tryp1** CTTGAACTCG AGGAATCCAT CAC---ATTC AACAGTGTAC GCTACCCTGT TCGTCTCCCT

**Tryp3** TTGGAACTAG CTGCTCCGGT GGA---ATTT AATGAGAAAC TCCAGCCAAT TAGGCTACCG

481 540

**Tryp2** GATGCCGATG AGGACATTGC TGATGGTACT GTTTTGAAGG TTTCTGGATG GGGGGATACT

**Tryp4** GAAATTAATG AATACCTTCC AAATGGAAGT AATCTGACTA TTTCCGGATG GGGAGATACG

**Tryp1** GAGAAAGATG ACGATGTCTA CGATGGTGCT CTTTTGCTTG TTTCCGGATG GGGTAATACC

**Tryp3** GAGCAGGATG AAGATGTGGA GGATGGAACA CCACTACTTG TTACCGGATG GGGAAATACG

541 600

**Tryp2** AAGAACCATC AGGAGTCCAA TTATCACGTC CGTGCTGTAT CAGTGCCGAA GGTTAATCAA

**Tryp4** AGAAATCCAA CTGAATCTGA GGAGCATCTC CGGGCTGTGT CAGTGCCAAA ATTGGAACAA

**Tryp1** CAGAACAGCC AGGAGAGCAA TAAACATTTG AGAGCTACTG TTGTGCCAAA ATACAATGAT

**Tryp3** CAGAATGCAC AGGAATCGCG AGAACAGCTA CGAGCTGCCA TTGTGCCCAA GAGCAATGAT

601 660

**Tryp2** TTAGAATGCC AAGCTAACTA CATTTTCGGA GCAATCATTT CGGAACAGAT GTTCTGTGCT

**Tryp4** TTAGAATGTA TAGCTAGTTA CTTATTTAGT GGCTTCATCA CAGACAGGAT GTTTTGCGCT

**Tryp1** GAACAATGCA ATAAGGCCTA CACCCAATAT GGCGGCATTA CAAAGACCAT GATCTGCGCT

**Tryp3** GAAGTCTGCA ACAAAGCCTA CGGGCAATTT GGCGGAATTA CGGCCAGGAT GATTTGTGCG

661 720

**Tryp2** GGTTACCAAG AAGGAGGAAA GGATTCTTGC CAAGGTGATT CTGGCGGTCC CGTCGTTGAC

**Tryp4** GGCTATCGTC AGGGTCAAAA GGATGCCTGC CAAGGCGACT CTGGTGGTCC TGTTCTGAAC

**Tryp1** GGATTTGAAG AGGGCGGCAA GGACGCCTGC CAAGGTGATT CCGGTGGTCC A---TTGACT

**Tryp3** GGACTCCCTG AAGGTGGCAA GGATGCCTGT CAAGGAGACT CTGGTGGCCC T---CTCGCC

721 780

**Tryp2** GAGAACAACG TTCAACATGG TGTAGTGTCT TGGGGTAAAG GTTGCGCCCT TCCAAGCTAT

**Tryp4** GATAATAACG TCCAGGTTGG AGTTGTTTCC TGGGGTCTTG ATTGCGCATT AGCAAGATAT

**Tryp1** CATGGAGATG TCCTTGTTGG TGTTGTTTCC TGGGGATTTG GATGTGCTCA ACCAAAATAT

**Tryp3** AGTGATGGTG TTCTCGTTGG TGTTGTATCC TGGGGTTATG GGTGTGCTGT CCGTGGGTAC

781 840

**Tryp2** CCTGGAGTCT ATGCAAAAGT ATCAGCTGTA AGGAATTGGA TTCGTGAAAT TTCAAATGTT

**Tryp4** CCTGGAGTCT ATGGAAGGAT CTCATCAGTC AGACAATGGA TCCGCGATAT CACTAATGTG

**Tryp1** CCCGGTGTTT ACTCCCGTGT TTCTTCCGTT CGCGAATGGG TTCATGAAGT CGTTGGTTTC

**Tryp3** CCAGGAGTTT ATTCGCGCGT TGCTTCAGTC AGGGATTGGA TCAATGCATC AACAAACATC

Figure S1: Multiple sequence alignment of *Lutzomyia longipalpis* putative trypsin cDNAs (Lltryp1 – accession number EF011106; Lltryp2 - accession number EF011107; LuloTryp3 – accession number EU124590; LuloTryp4 - accession number EU124582). Identical DNA bases are highlighted in red. Sites of primers for trypsin 1 dsRNA construction are boxed.
